# Pulmonary Hypertension in General Cardiology Practice

**DOI:** 10.5935/abc.20190188

**Published:** 2019-09

**Authors:** Daniela Calderaro, José Leonidas Alves Junior, Caio Júlio César dos Santos Fernandes, Rogério Souza

**Affiliations:** 1 Instituto do Coração - Hospital das Clínicas HCFMUSP - Faculdade de Medicina - Universidade de São Paulo, São Paulo, SP - Brazil

**Keywords:** Hypertension, Pulmonary, Pulmonary Heart Disease, Echocardiography/methods, Pulmonary Disease, Chronic Obstructive, Pulmonary Emphysema, Pulmonary Fibrosis

## Abstract

The finding of pulmonary hypertension (PH) by echocardiography is common and of concern. However, echocardiography is just a suggestive and non-diagnostic assessment of PH. When direct involvement of pulmonary circulation is suspected, invasive hemodynamic monitoring is recommended to establish the diagnosis. This assessent provides, in addition to the diagnostic confirmation, the correct identification of the vascular territory predominantly involved (arterial pulmonary or postcapillary). Treatment with specific medication for PH (phosphodiesterase type 5 inhibitors, endothelin receptor antagonists and prostacyclin analogues) has been proven effective in patients with pulmonary arterial hypertension, but its use in patients with PH due to left heart disease can even be damaging. In this review, we discuss the diagnosis criteria, how etiological investigation should be carried out, the clinical classification and, finally, the therapeutic recommendations for PH.

## Introduction

In the cardiologist's routine, the echocardiographic finding of pulmonary hypertension (PH) is extremely common. PH on echocardiogram can be identified in up to 2.8% of the general population^1^ and in more than half of the patients with heart failure. It is estimated that almost 100% of the individuals with symptomatic mitral regurgitation and the majority of those with major aortic stenosis show some degree of increased systolic pulmonary artery pressure.^2,3^

The diagnosis of PH has major prognostic implications, both when it is attributable to cardiovascular diseases,^4^ and to pumonary diseases,^5^ or even in isolated pulmonary vascular involvement. Unfortunately, it is common to initiate specific treatment indiscriminately for PH patients based only on echocardiographic data, which, in some cases, can increase mortality.^6^ It is essential to perform detailed investigation to confirm the diagnosis and comprehension of the mechanisms predominantly involved in PH and, thus, determine the correct therapeutic approach. These topics will be discussed in this review.

### Definition

Traditionally, PH is defined through invasive hemodynamic monitoring, with a recent review of the value assumed as pathological. Since 1973, the diagnosis of PH was arbitrarily made when mean arterial pulmonary pressure (mPAP) was equal to or greater than 25 mmHg.^7^ However, recent data have shown that, even with lower mPAP values, there is an increase in mortality rates.^8^ Therefore, on December 2018, a consensus resulting from the 6th World Symposium on Pulmonary Hypertension was published, which redefined PH to the situation in which mPAP is higher than 20 mmHg and pulmonary vascular resistance is greater than or equal to 3 Woods units.^9^

When, in the presence of this level of pulmonary pressure, the pulmonary artery occlusion pressure (PAOP) is equal to or less than 15 mmHg, circulatory impairment begins to occur in the pulmonary circulation, either due to pulmonary arterial hypertension (PAH), or to pulmonary thromboembolism or pulmonary parenchymal disease. If the PAOP is higher than 15 mmHg, the PH is considered postcapillary. In this case, increased pressure in the pulmonary arterial territory is due to retrograde transmission of increased left atrial hydrostatic pressure into the pulmonary veins and pulmonary capillaries and, ultimately, into the pulmonary arterial circulation. There are situations in which the PAOP is above 15 mmHg, but this fact does not seem suficient to justifify the severity of mPAP increase. These patients display a pulmonary vascular resistance greater than 3 Woods units and, usually, the diastolic pulmonary gradient is higher than 7 mmHg (GDP - the difference between the pulmonary artery diastolic pressure and the pulmonary capillary pressure).^10^

This condition is called combined pre- and post-capillary PH. This hemodynamic profile can occur in patients with left heart disease and pulmonary vascular remodelling secondary to chronic congestion, but can also be seen in severely hypervolemic patients with PAH, and reverse Bernheim effect (when PH is so severe that results in the interventricular septum bulging toward the left ventricle, thus increasing the left ventricular pressure and, therefore, the PAOP).^11^

Right heart catheterization is indispensable for the diagnosis of PH, with a morbidity of 1.1% and mortality of 0.055%, in experienced centers.^12^ On the other hand, its implications for the accurateness of the diagnosis are overwhelming. In a study conducted at a referral center for PH in Brazil,^13^ out of the 384 patients with echocardiography suggestive of PH undergoing right heart catheterization, only 78.6% actually had a mPAP ≥ 25 mmHg. Thus, if the diagnosis of PH is based on echocardiography alone, mistakes may occur in more than 20% of the cases. Moreover, in the same study, among the patients wih PH, 18.3% had post-capillary PH (PAOP > 15 mmHg), which has direct implications for the treatment. Without the catheterization, a quite considerable number of patients would be inadequately diagnosed and treated.

### Classification of pulmonary hypertension

The current classification of PH takes into account data of clinical presentation, pathophysiology, anatomopathological findings and hemodynamic parameters,^7,9,11^ and proposes a division into 5 different groups (Table 1). It should be highlighted that, since 2003, the terms “primary” and “secondary” PH are no longer listed in the WHO consensus.

**Table 1:** Classification of pulmonary hypertension^7,9^

Group 1	Pulmonary Arterial Hypertension
Group 2	Pulmonary hypertension due to left heart disease
Group 3	Pulmonary hypertension due to Pulmonary Disease and/or Hypoxia
Group 4	Chronic thromboembolic pulmonary hypertension and other diseases of pulmonary artery obstruction
Group 5	Pulmonary hypertension with unclear multifactorial mechanisms

#### Group 1

Patients with PAH. These are the patients with idiopathic PAH, heritable PAH, associated with HIV infection, connective tissue disease, portal hypertension, drugs or congenital heart diseases. Pulmonary veno-occlusive disease (PVOD) and pulmonary capillary hemangiomatosis (PCH) are also categorized as Group 1.^14^ Schistosomiasis-associated PH has a major epidemiological relevance in Brazil, and is included in this group.^15-18^ In Group 1 patients, the catheterization reveals pattern of pre-capillary HP (PAOP ≤ 15 mmHg), and does not show significant pulmonary heart disease or chronic thromboembolic HP. The histological findings are vasoconstriction, vascular remodelling with plexiform lesions and microthrombosis in the pulmonary vasculature.^19^ Studies with specific medication for the treatment of PH mainly comprise this group.

#### Group 2

Patients with HP due to left heart disease: valvular disease, left ventricular diastolic or systolic dysfunction. These patients have hemodynamic patterns of post-capillary hypertension. In the cases where combined post-capillary PH with a pre-capillary component is observed, the prognosis is worse than that for patients with isolated post-capillary PH.^20^ The identification strategy of this hemodynamic profile can be sensitized by performing fluid challenge during catheterization. Elderly patients with metabolic syndrome, atrial fibrillation or changes in the left heart, revealed by echocardiography, have a high probability that their HP will be due to the post-capillary component. In these situations, if the PAOP is ≤ 15 mmHg and > 12 mmHg, a new fluid challenge during catheterization should be considered.^21^ The administration of 500mL of saline solution within 5 minutes is recommended, being the post-capillary component assumed when the PAOP, measured immediately after the fluid challenge, is greater than 18 mmHg.^21^ This is the most prevalent type of PH worldwide.^22^

#### Group 3

Patients with HP due to pulmonary disease and/or hypoxia. For instance: chronic obstructive pulmonary disease, interstitial lung disease, obstructive sleep apnea, high altitude exposure. The hemodynamic pattern is that of pre-capillary HP.^9^

#### Group 4

Patients with chronic thromboembolic pulmonary hypertension (CTEPH) or diseases of pulmonary artery obstruction such as arteritis, neoplasms, or congenital pulmonary artery stenosis, with hemodynamic pattern of pre-capillary PH.^23^ The aim of the treatment in this population is to restore blood flow to the obstructed vascular territories.

#### Group 5

Patients with HP and unclear multifactorial mechanisms, as in the cases of renal failure, sarcoidosis, myeloproliferative disorders and hemolytic anemia.^7^

### Diagnostic evaluation of pulmonary hypertension

The diagnostic suspicion is based on unspecified symptoms (dyspnea to effort and/or syncope), not always accompanied by signs suggestive of PH or right ventricular dysfunction (hyperphonesis of the second heart sound, tricuspid systolic murmur, jugular stasis, hepatomegaly and lower limb edema). Considering these findings, the non-invasive test of choice to begin the investigation is the transthoracic echocardiography.^19^

The interval between the symptom onset and the diagnosis of PH is about two years, which hinders early treatment.^7^

**Figure 1:**
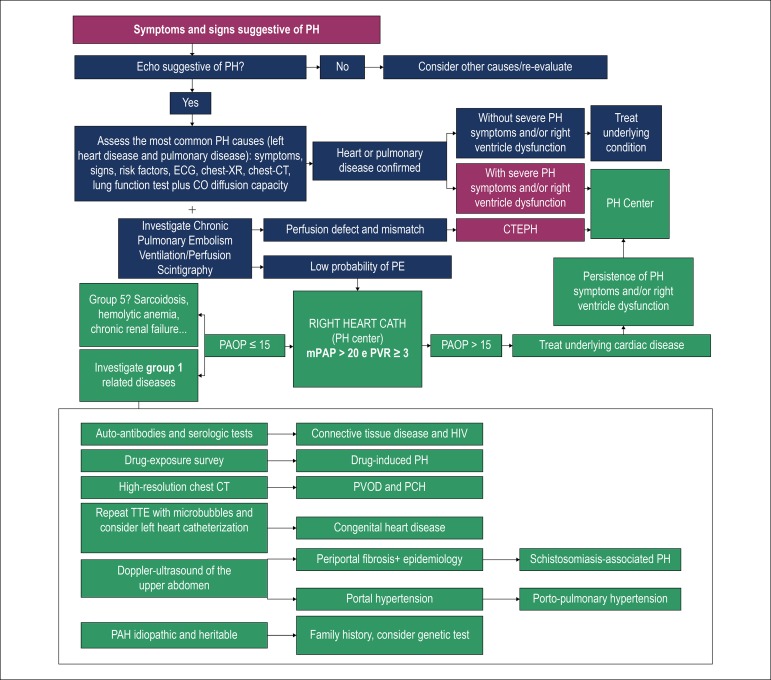
Diagnostic algorithm (adapted from Alves-Jr, et al.19). DLCO: Diffusing capacity of the lungs for carbon monoxide; V/Q scintigraphy: Ventilation and pulmonary perfusion scintigraphy; CTEPH: PH due to chronic pulmonary thromboembolism; RHC: Right heart catheterization; HRCT: High-resolution CT; TTE: Transthoracic doppler echocardiogram; PVOD: Pulmonary veno-occlusive disease; PCH: pulmonary capillary hemangiomatosis.

The investigation should begin by searching for the most frequent causal factors: left heart disease, lung disease or pulmonary thromboembolism. Only after excluding these conditions, should the presence of PAH be considered, as proposed in the algorithm (Figure 1).

### CHEST X-Ray

It can show prominence of the pulmonary artery trunk, as well as of the right (> 16 mm) and/or left (> 18 mm) branches, increased right chambers (bulging of the right mediastinal contour, boot shaped heart and filling of retrosternal space).^24^ These changes are usually more marked only in advanced stages of the disease.

### Electrocardiography

Traditionally, the electrocardiography shows signs of overload in the right chambers - axis shift to the right and P-wave pulmonale (p ≥ 2.5 mm in DII); as in the X-ray, the electrocardiographic changes are more evident in the stages when there is cardiac structure repercussion. In up to 13% of the cases, the EGC is normal.^25^

### Chest CT

Computed angiotomography of the chest plays an important role in the differential diagnosis of PH and its classification, helping to investigate diseases that affect the pulmonary parenchyma and chronic thromboembolism. It can also increase the suspicion of the diagnosis of pulmonary veno-occlusive disease. Computed tomography findings of increased pulmonary artery diameter, of secondary importance compared to echocardiography, are also suggestive of PH and, thus, can be used in indirect screening. This measurement of the pulmonary artery diameter exhibits quite high specificity for the presence of HP when the diameter is greater than 33.2 mm.^26^

### Ventilation/Perfusion Scintigraphy

Ventilation/perfusion scintigraphy is essential for CTEPH screening due to its high sensitivity (96-97%), combined with a specificity of 90-95%, while chest CT angiography can have a sensitivity of up to 50%.^27^ Nevertheless, dual-energy computed tomography has been shown to have the same sensitivity and specificity for CTEPH than scintigraphy.^28^

### Echocardiography

It is the best non-invasive screening method for PH, but it does not establish the definitive diagnosis, nor does it make a clear distinction between the different PH groups (Table 1). Echocardiography allows for the assessment of pulmonary artery systolic pressure - according to the direct measure of the tricuspid regurgitation speed and the estimate of right atrial pressure - in addition to assessing the right and left ventricular functions. Besides the cavity dimension, specifically in relation to right ventricular assessment, several parameters are used, such as TAPSE - tricuspid annular plane systolic excursion, the area difference between RV diastolic and systolic areas, called right ventricular fractional area change (FAC), myocardial performance index (MPI), left ventricular ejection fraction (LVEF) as measured by two-dimensional (2D) and three-dimensional (3D) echocardiography, DTI-derived tricuspid lateral annular systolic velocity (S'-wave) and longitudinal strain.^29^

### Prognosis Assessment

In spite of all the advances of the last two decades, PAH remains a high-mortality disease (an approximate 25% mortality rate at 3 years, according to recent registries).^30^ There are several markers associated with the prognosis of PAH which can be used in clinical practice for therapeutic follow-up of patients under specific therapy.^31^ Based on the results of these prognostic markers, it is possible to decide on stabilisation of the medication or therapeutic escalation. Table 2 shows the values defined as better, intermediate or worse prognosis of each marker.^11^ Recent researches have indicated the possibility of using noninvasive prognostic markers in follow-up assessments (BNP, 6-minute walk test and functional class), with a good survival prediction.^32^

**Table 2:** Risk assessment in pulmonary arterial hypertension - adpted from the European guidelines on pulmonary hypertension published in 2015^11^

Prognostic marker/RisK	Low risk (Estimated mortality < 5% year)	Intermediate risk (Estimated mortality 5-10% year)	High risk (Estimated mortality > 10% year)
Signs of heart failure	Absent	Absent	Present
Progression of symptoms	No	Slow	Rapid
Syncope	No	Occasional	Frequent
Functional class	I, II	III	IV
6MWD	> 440 m	165-440 m	< 165 m
Cardiopulmonary exercise testing	Peak VO_2_ > 15 ml/min/kg (> 65% pred.) VE/VCO_2_ slope < 36	Peak VO_2_ 11-15 ml/min/kg (35-65% pred.) VE/VCO_2_ slope 36-44.9	Peak VO_2_ < 11ml/min/kg (< 35% pred.) VE/VCO_2_ slope ≥ 45
BNP levels	BNP < 50 ng/L NT-proBNP < 300 ng/L	BNP 50-300 ng/L NT-proBNP 300-1400 ng/L	BNP > 300 ng/L NT-proBNP > 1400 ng/L
Imaging	RA area < 18 cm^2^ No pericardial effusion	RA area 18-26 cm^2^ No or minimal pericardial effusion	RA area > 26 cm^2^ Pericardial effusion
Hemodynamics	RAP < 8 mmHg CI ≥ 2.5 l/min/m^2^ SvO_2_ > 65%	RAP 8-14 mmHg CI > 2-2.5 l/min/m^2^ SvO_2_ 60-65%	RAP > 14 mmHg CI < 2.0 l/min/m^2^ SvO_2_ < 60%

VO_2_: consumo de oxigênio; VCO_2_: liberação de dióxido de carbono; Slope VE/VCO_2_: equivalente respiratório para o dióxido de carbono; BNP: peptídeo natriurético cerebral; NT pro BNP: fragmento N-terminal do pró BNP; AD: átrio direito; In.C: índice cardíaco; SVO_2_: saturação venosa mista de oxigênio

### Treatment

#### Pulmonary Arterial Hypertension (Group 1)

After definition of the diagnosis, initiation of treatment can be considered. It should be highlighted that, in patients with PH associated with HIV infection, or in patients with systemic lupus erythematosus or mixed connective tissue disease, it is necessary to treat the underlying disease, which may be sufficient to treat PAH.^24^

General measures for PH include: physical rehabilitation, avoiding excessive physical activity, psychosocial support, avoiding pregnancy, immunization against influenza and pneumococcal infection. Treatment with diuretics, O_2_therapy and digoxin are considered supportive therapy. Oral anticoagulant therapy may be considered in patients with IPAH, HPAH and anorexigen-induced PAH.^19^

Calcium-channel blockers are recommended only in cases of PAH with a positive acute vasoreactivity test. This test is performed with nitric oxide (NO) inhalation (10-80 ppm) for 10 minutes, and is indicated in the cases of idiopathic, heritable or drug-induced PAH.^7^ Epoprostenol, iloproste or adenosina can also be used. The test is deemed positive when, after the vasodilator infusion, the mPAP decreases to less than 40 mmHg, with a variation of at least 10 mmHg, in association with a maintained or increased cardiac output. This assessment allows for identification of the subpopulation with PAH (about 10%) whose main pathophysiological mechanism is pulmonary vasoconstriction, with a better medium- and long-term prognosis.^33^ High doses of calcium-channel blockers should only be used in this situation, because they worsen the prognosis of patients who do not respond to the test.

Group 1 PAH-specific therapy arose from the decade of 1990 on. These medications target three pathophysiological pathways of the disease: the prostacyclin pathway, the nitric oxide pathway, and the endothelin pathway (Table 3).

**Table 3:** Specific drugs available for PH treatement (modified from Galiè N, et al.^11^)

Pathophysiological pathways	Class	Drug
Endothelin	Endothelin Receptor Antagonists 1	Ambrisentan
		Bosentan
		Macitentan
Nitric Oxide	Phosphodiesterase type5 inhibitors	Sildenafil
		Tadalafil
		Vardenafil
	Soluble Guanylate Cyclase Stimulants	Riociguat
Prostaglandins	Prostacyclin	Epoprostenol
	Prostacycline Analogues	Iloprost
		Treprostinil
		Beraprost
	Selective IP receptor agonists	Selexipag

Endothelin Receptor Antagonists 1 (ambrisentan, bosentan and macitentan) and phosphodiesterase type 5 inhibitors (nitric oxide pathway - sildenafil and tadalafil) are more recurrent in Brazil, and are often used as monotherapy or in combination as first line in the treatment of pulmonary arterial hypertension.^15,34^

Prostanoids were the first class of medication used in pulmonary arterial hypertension and, in addition to improving morbidity and exercise capacity, epoprostenol was the ony drug to show survival improvement in a clinical randomized trial.^35^ This drug class should always be considered for patients with FC-IV symptoms.^35,36^

**Figure 2:**
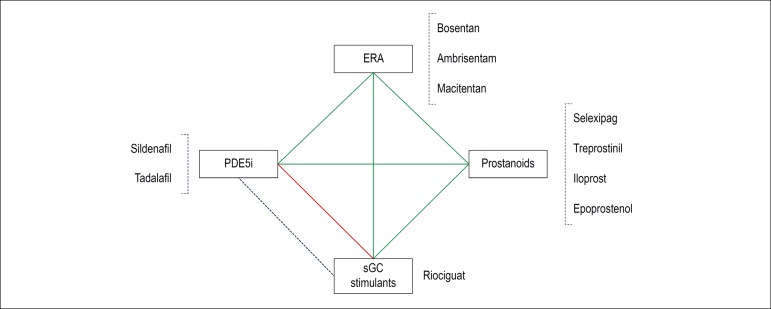
Pathophysiological pathways in pulmonary hypertension and specific therapy. Green lines: possible combinations; Red lines: Not recommended combination; Blue dotted line: Potential for substitution therapy, within the same pathway. ERA: endothelin receptor antagonist; PDE5i: Phosphodiesterase type 5 inhibitor; sGC: Soluble Guanylate Cyclase. Modified from Dos Santos Fernandes CJC, et al.^37^

In cases of progressive disease, or even in cases where prognostic stratification in the initial approach is already suggestive of high risk, the use of combined therapy should be considered.^36^ Drugs that act in different pathways should be combined (Figure 2).^37^ Once there are no more possibilities of clinical management of PAH, atrial septostomy or even lung transplantation should be considered.^11^

#### Pulmonary hypertension due to left heart disease (Group 2)

Pulmonary hipertension in patients with left-sided cardiomyopathy is the most frequent form of PH. It is difficult to establish its exact prevalence because most reports are based on echocardiographic findings, with no confirmation of diagnosis through catheterization. Pulmonary congestion, due to retrograde transmission of elevated filling pressures in the left heart, determines directly an increase in pulmonary artery blood pressure; what happens is that, in adittion to this passive mechanism, congestion is also associated with the activation of neuro-hormonal mechanisms and eventually with vascular remodelling. Thus, a pre-capillary component can arise, combined with the post-capillary component, characteristic of left heart disease. In this context, there might be rationale for the use of specific PAH medication; however, until now, there is no significant evidence of the benefits of such approach, as we shall see.

### Epoprostenol

Twenty years ago, Califf et al.^38^ carried out a study to assess the impact of epoprostenol in 471 patients with advanced heart failure (HF) (LVEF < 25%, NYHA functional class III or IV and optimized therapy for those times: ACE inhibitors/diuretics/digitalis; many in need of inotropic support).^38^ In spite of clear improvement of hemodynamic parameters with the use of epoprostenol (increased cardiac index, decreased pulmonary capillary pressure and reduced systemic and pulmonary vascular resistance), the study was precociously interrupted due to increased mortality within 6 months: 48% vs 37%. There was no significant differences in symptoms, quality of life or walking distance. Minor studies have shown conflicting data of clinical and/or hemodynamic improvement, but they had no power to assess mortality.^39^ Until today, the use of epoprostenol (or the prostanoid analogs, i.e., iloprost, treprostinil, beraprost, or even selexipag, a specific IP-receptor agonist, which acts on the same pathway as prostaglandins) is not recommended for group 2 PH.

### Endothelin receptor antagonists

Blockade of ETA receptors in patients with group 2 PH can be useful not only for the well-known effects on pulmonary circulation, but also because of its potential direct benefits to the systemic circulation and myocardium. In 1998, Sütsch etal.^40^ observed an increase in cardiac output, decrease in pulmonary capillary pressure and severe systemic and pulmonary vascular resistance in a small number of patients with advanced HF, randomized to receive bosentan 1 g (a non-selective inhibitor of endothelin receptors A and B).^40^ Four years later, in the ENABLE study, 1,613 patients with NYHA functional class III or IV heart failure due to severe left ventricular dysfunction (EF < 35%) received bosentan (125 mg 2X/day) or placebo; there was no difference in the primary outcome (death or hospitalization due to heart failure) and there was higher fluid retention in the group who had received the medication.^41,42^ In the HEAT study, 157 patients with FC III HF (and cardiac index ≤ 2.6 L/min.m^2^ and PCP ≥ 12 mmHg) were randomized to receive placebo or darusentan (a selective inhibitor of endothelin receptor A). The group who received darusentan showed increased cardiac index (the study's primary outcome) and decreased systemic resistance after 3 weeks of treatment, with no changes in pulmonary vascular resistance, pulmonary artery pressure or pulmonary capillary pressure (also a primary endpoint). However, the patients who received the medication had more clinical decompensation, notably the highest dose groups.^43^ The same medication was studied again in 642 patients with FC II-IV HF and LVEF < 35%. There was no right heart catheterization monitoring. Patients were randomized for placebo or darusentan (5 doses) for 6 months. The primary outcome of the study was LV diastolic volume changes measured using cardiac resonance imaging. The selective inhibitor of endothelin receptor had no impact on cardiac remodelling or in the clinical parameters, again with a tendency towards higher decompensation of heart failure with moderate to higher doses of the medication studied.^44^

Another study, with bosentan, assessed the impact of the medication in high dosage (500 mg 2x/day) and for an extended period of time (26 weeks) in 370 patients with advanced HF, in FC III or IV and with LVEF < 35%. The primary outcome was clinical worsening. The study was interrupted precociously due to the high incidence of hepatotoxicity, so that less than half of the case reports had completed the 26 weeks of follow-up. This data is important since, in general, there was no difference between the bosentan and placebo groups, but for those patients who completed the 6 months of follow-up, the use of bosentan was associated with clinical improvement. We do not have the hemodynamic data of the mentioned study.^45^

So far we have discussed studies that analysed patients with LV systolic dysfunction, and mostly, though not entirely, PH registries (not always by right heart catheterization). If on one hand there seems to be hemodynamic improvement in the early stage of endothelin receptor inhibitor therapy, on the other hand, there may be clinical decompensation, probably due to hydrosaline retention. In the medium-term, sustained usage can bring clinical benefits, as suggested in the latest study discussed above.^45^ Recently, the results of the MELODY-1 study (Macitentan in subjects with combined prE- and post-capiLlary PH due to left ventricular DYsfunction) can be incorporated into the discussion on the role of inhibitors of endothelin receptors in the treatment of patients with PH due to left ventricular dysfunction.^46^ Although this is a pilot study, with small casuistry, it is the first study carried out from hemodynamic confirmation of group 2 PH to randomization. The authors limited the study to patients with group 2 PH, along with the hemodynamic criteria of post- and pre-capillary componentes of PH (pulmonary vascular resistance > 3 Wood units and diastolic pulmonary gradient > 7 mmHg). A total of 63 patients were randomly assigned to macitentan or placebo. There was more fluid retention or clinical worsening in the macitentan group, especially due to the first criterion: 22.6% X 12.5%. After 12 weeks, no difference was observed between the groups in relation to the hemodynamic parameters, BNP or 6 min walk test. Although the authors selected, among the patients with HF, the subgroup more likely to benefit from specific medication for PAH, they also observed worsened evolution when they used it,^47^ which is nowadays one of the strongest evidence against the use of inhibitors of endothelin receptors in group 2 PH.

### Phosphodiesterase type 5 inhibitors

In 2007, Lewis et al.^48^ randomized 34 patients with FC II-IV heart failure and LVEF ≤ 40% to receive sildenafil or placebo for 3 months. The patients who received sildenafil improved HF functional class, aerobic capacity, the distance in the 6-minute walk test and showed significant reduction in pulmonary vascular resistance in relation to the basal values, with no increase in pulmonary capillary pressure or change in cardiac index.^48^ Meanwhile, encouraging case reports were published on hemodynamic improvements through treatment with sildenafil in candidates for cardiac transplantation, with reduced pulmonary vascular resistance and cardiac outcome improvement.^49^

Other small studies have also demonstrated hemodynamic benefits in patients with LV systolic dysfunction who received silnadefil, even after the first dose.^50^ Nonetheless, there are no data of randomized multicenter studies available to establish the impact of sildenafil on patients with LV systolic dysfunction. In the Sildenafil Versus Placebo in Chronic Heart Failure (SilHF) study, still in progress, patients with HF and Group 2 PH (echocardiographic criterion) and LVEF < 40% are randomized to receive sildenafil 40 mg 3x/day or placebo.^51^ This will be an important study, though the lack of hemodynamic monitoring through right catheterization may be a problem for the interpretation of the results.

The impact of sildenafil when compared with placebo throughout 12 weeks was tested in patients with heart failure with preserved ejection fraction. In this multicenter study, 216 patients were randomized and there was no difference in the primary outcome (peak consumption of O_2_) or in the clinical Picture.^52^ We do not have the data on right heart catheterization and therefore we do not know the exact number of patients in group 2 PH.

Finally, the subgroup of patients with valvular heart disease deserves special attention. Most patients with significant aortic valvular disease and almost all patients with symptomatic mitral insufficiency have PH. Even after correction of the valvular disease, some patients remain with PH and others who did not have PH before surgical treatment can develop the disease evolutionarily. Recently, the impact of sildenafil in patients with residual PH, after heart valve surgery, was assessed in a multicenter randomized study.^3^ A total of 200 patients with a mean pulmonary arterial pressure ≥ 30 mmHg and with no signficant valve injury were randomized to receive sildenafil 40 mg every 8 hours or placebo, for 6 months. The composite outcome of death, admission to hospital for decompensated heart failure or functional worsening occurred more often in the sildenafil group compared with the placebo group (OR 0.39; CI 0.22-0.67; p < 0.001), mainly at the expense of more admissions for decompensed HF. It is important to highlight that, although the etiology of the cardiomyopathy was valvular disease, and only patients with no significant residual valvular injury had been randomized, the hemodynamic data indicate characteristics of group 2 PH, with combined pre- and post-capillary PH; in a little more than half of the patients (57%), pulmonary resistance was greater than 3 Wood units.

Thus, althought early data from small case reports performed in single-centers encourage the use of sildenafil in grupo 2 PH, there is no evidence supporting its routine recommendation.

### Riociguat

Riociguat acts on the same pathway as phosphodiesterase type 5 inhibitors, directlty stimulating guanylate cyclase, in addition to having an established role in the management of patients with PH due to chronic pulmonary thromboembolism (group 4) and in pulmonary arterial hypertension. Its role in group 2 PH has been recently tested in the LEPHT study.^53^ The patients had symptomatic HF, with LFEF ≤ 40% and PH measured by right heart catheterization. A total of 201 patients were radomized to receive riociguat (3 different doses) or placebo. There was no difference in the mean pulmonary artery pressure between the groups (primary endpoint), but the group who received riociguat 2 mg three times daily showed increased cardiac index and reduced systemic and pulmonary vascular resistance. No difference was observed in relation to the functional class or in the composite outcome of death or admission for decompensated heart failure.

Thus, so far, there is no indication for the routine use of any of the specific medications for group 1 PH in individuals with group 2 PH. In selected cases, when, after optimization of cardiomyopathy, with special attention to volemia, PH remains with pre-capillary component apparent on cardiac catheterization, the decision upon the use of specific medication should be individualized, in a reference center, preferably, in the context of clinical study to deliver more evidence.

### Pulmonary hypertension due to Pulmonary Disease and/or Hypoxia (Group 3)

It is considered the second most common cause of PH and the pulmonary diseases most commonly associated with PH are: chronic obstructive pulmonary disease (COPD), interstitial pulmonary disease and combined pulmonary fibrosis and emphysema.^54^

Although there is high prevalence of increased pulmonary artery pressure in patients with chronic pulmonary diseases, only a small minority of these patients present with severe PH, characterized by mPAP > 35 mmHg. In some patients with pulmonary disease and HP, especially in patients with mild pulmonary disease, but with severe PH, it may be difficult to determine whether PH is caused by pulmonary disease or related to concomitant pulmonary vascular disease.^55^ Until now, there is no evidence that the specific medications used in PAH are beneficial for the treatment of Group 3 PH, and patients with suspected associated vascular disease should be referred to reference centers.

### Chronic thromboembolic pulmonary hypertension (CTEPH) and other diseases of pulmonary artery obstruction (Group 4)

CTEPH is the main disease of group 4, and is characterized by chronic obstruction of pulmonary artery and vascular remodelling due to pulmonary thromboembolism. It is the only potentially curable form of PH, once effective pulmonary thromboendarterectomy is perfomed. For this reason, it is always necessary to investigate chronic pulmonary thromboembolism in patients with PH, and refer the diagnosed cases to a reference center.^56^ In cases of contraindicated surgery or persistent PH after surgery, there is evidence in favor of the use of riociguat.^57^ Macitentan also provides clinical and hemodynamic benefits for patients with CTEPH with no surgical indication.^58^ Full anticoagulation is always recommended, even after surgery; diuretic and oxigen are indicated in case of heart failure and hypoxemia, respectively.^11^ should be considered for patients who are not candidate for surgical intervention.^59^

### Pulmonary hypertension with unclear multifactorial mechanisms (Group 5)

Group 5 includes several diseases which may behave similarly to other groups, but whose mechanisms associated with the development of PH are not clear yet. Thus, treatment is heterogeneous and essentially focused on the underlying disease.^60^

## Conclusion

PH is a complex and heterogeneous condition, often wrongly diagnosed when based only on echocardiographic data. For patients with Grupo 1 PH, the use of specific therapeutic approaches are recommended. Unfortunately, for the most common forms of PH: group 2 (cardiac cause) or group 3 (respiratory causes) routine use of specific therapeutic is not indicated. The complexity of the assessment of patients with PH reinforces the need for these patients to be followed in centers with expertise in pulmonary circulation, where multidisciplinary approach allows for optimization of existing resources and treatment adequacy to current guidelines.
